# The Gastroprotective Effect of Menthol: Involvement of Anti-Apoptotic, Antioxidant and Anti-Inflammatory Activities

**DOI:** 10.1371/journal.pone.0086686

**Published:** 2014-01-21

**Authors:** Ariane Leite Rozza, Felipe Meira de Faria, Alba Regina Souza Brito, Cláudia Helena Pellizzon

**Affiliations:** 1 Pharmacology Department, Biosciences Institute, Universidade Estadual Paulista - UNESP, Botucatu, Brazil; 2 Pharmacology Department, Faculty of Medical Sciences, University of Campinas - UNICAMP, Campina, Brazil; 3 Department of Structural and Functional Biology, Biology Institute, University of Campinas - UNICAMP, Campinas, Brazil; 4 Morphology Department, Biosciences Institute, UNESP – Universidade Estadual Paulista - UNESP, Botucatu, Brazil; Charité-University Medicine Berlin, Germany

## Abstract

The aim of this research was to investigate the anti-apoptotic, antioxidant and anti-inflammatory properties of menthol against ethanol-induced gastric ulcers in rats. Wistar rats were orally treated with vehicle, carbenoxolone (100 mg/kg) or menthol (50 mg/kg) and then treated with ethanol to induce gastric ulcers. After euthanasia, stomach samples were prepared for histological slides and biochemical analyses. Immunohistochemical analyses of the cytoprotective and anti-apoptotic heat-shock protein-70 (HSP-70) and the apoptotic Bax protein were performed. The neutrophils were manually counted. The activity of the myeloperoxidase (MPO) was measured. To determine the level of antioxidant functions, the levels of glutathione (GSH), glutathione peroxidase (GSH-Px), glutathione reductase (GR) and superoxide dismutase (SOD) were measured using ELISA. The levels of the pro-inflammatory cytokines tumor necrosis factor-α (TNF-α) and interleukin-6 (IL-6) and the anti-inflammatory cytokine interleukin-10 (IL-10) were assessed using ELISA kits. The menthol treated group presented 92% gastroprotection compared to the vehicle-treated group. An increased immunolabeled area was observed for HSP-70, and a decreased immunolabeled area was observed for the Bax protein in the menthol treated group. Menthol treatment induced a decrease in the activity of MPO and SOD, and the protein levels of GSH, GSH-Px and GR were increased. There was also a decrease in the levels of TNF-α and IL-6 and an increase in the level of IL-10. In conclusion, oral treatment with menthol displayed a gastroprotective activity through anti-apoptotic, antixidant and anti-inflammatory mechanisms.

## Introduction

Gastric ulcers, the most common gastric disease, are considered to be a global health problem, affecting approximately 14.5 million people worldwide [1]. The current treatments for patients suffering from gastric ulcers include antacids, histamine H_2_ receptor antagonists and proton pump inhibitors. The main therapeutic target of these classes of medications is the secretion of gastric acid. However, there is growing evidence that the ideal antiulcer compound should inhibit gastric secretion but should also have multiple effects. In addition to an antisecretory effect, an effective treatment must possess antioxidant, anti-apoptotic and anti-inflammatory activities [2].

Recent research by our group demonstrated the gastroprotective effect of the cyclic terpene (-)-menthol, which mainly affects mucus secretion and prostaglandin E_2_ (PGE_2_) production [3]. The present study was undertaken to further investigate the mechanism of menthol action, analyzing the possible gastroprotective anti-apoptotic, antioxidant and anti-inflammatory effects of menthol against ethanol-induced gastric ulcers in rats.

## Materials and Methods

### 2.1. Menthol

(-)-Menthol (catalog #63660, >99% purity) was purchased from Sigma-Aldrich. (St. Louis, MO, USA).

### 2.2. Animals

Male Wistar rats weighing 200–250 g from the Central Animal House of UNESP were fed a certified diet with free access to tap water under standard light-dark cycles (12 h dark/12 h light), humidity (60±1%) and temperature (21±2°C). Prior to experimentation, all rats were fasted for 16 hours and housed in cages with raised floors of a wide mesh to prevent coprophagy. No anesthetic procedure was done before the oral administrations. After the experiment, the rats were euthanized in a pre-saturated CO_2_ chamber. All efforts were done to minimize animal suffering. All experimental protocols followed the recommendations of the Canadian Council on Animal Care and were approved by the UNESP Institutional Animal Care and Use Committee (permit number 221-CEEA, 2010).

### 2.3. Ethanol-induced gastric ulcers

Male Wistar rats that had been fasted for 16 h were distributed into three groups (n = 7). The rats were then orally dosed with the vehicle (8% Tween 80, 10 mL/kg, Sigma-Aldrich, St. Louis, MO, USA), carbenoxolone (100 mg/kg, Sigma-Aldrich, St. Louis, MO, USA) or menthol (50 mg/kg). The dose of the menthol was selected based on a previous dose-response assay; the dose of 50 mg/kg was shown to be the lowest effective dose [3]. After 1 h, the animals received an oral dose of 1 mL of absolute ethanol. One h after ethanol treatment, the rats were euthanized and their stomachs were removed [4]. The stomachs were then opened along the greater curvature and washed. The stomachs were scanned and the ulcer area (mm^2^) was measured using AVSoft BioView software.

The stomach samples were scrapped after the scans and frozen at −80°C until biochemical analyses were performed. Histological slides were prepared from intact samples.

### 2.4. Histological analysis

Samples of the stomach of each rat were fixed in alfac solution (85% alcohol 80, 10% formalin and 5% acetic acid) and processed in a paraffin tissue processing machine. Sections of the stomach were cut to a thickness of 5 µm and stained with hematoxylin and eosin (HE) for histological evaluation. Neutrophils were counted on the HE-stained slides. The slides that were not stained were submitted to immunohistochemical analysis.

### 2.5. Immunohistochemical analysis

Tissue section slides were deparaffinized, rehydrated and immunostained with peroxidase. Non-specific reactions were blocked with H_2_O_2_ and goat serum prior to incubation with the appropriate specific antibody. The sections were rinsed with phosphate-buffered saline (PBS, 0.01 mol/L, pH 7.4) and incubated with a secondary antibody (Avidin-Biotin Complex ABC kit, Erviegas, São Paulo, SP, Brazil). The sections were then washed with phosphate buffer solution (PBS), the ABC reagent was applied, and the reaction was carried out in a 3,3′-diaminobenzidine tetrahydrochloride (DAB) solution containing 0.01% H_2_O_2_ in PBS. After immunostaining, the sections were lightly counterstained with hematoxylin and the immunolabeled cells were observed and photographed under a Leica microscope using Leica QWin Software (Leica, Wetzlar, Hessen, Germany). For the control reactions, the slides were processed without the primary antibody or the secondary antibody. Positive signals in the immunohistochemical staining were observed as brown-staining areas under light microscopy. The slides were stained with antibodies against heat-shock protein 70 (HSP-70, 1∶500 dilution) and Bax (1∶200 dilution) (Santa Cruz Biotechnology, Dallas, Texas, USA). Ten fields in each slide were photographed and the brown-stained areas were measured (µm^2^) using AVSoft BioView software (AVSoft Softwares Laboratoriais, Campinas, SP, Brazil).

### 2.6. Preparation of samples for biochemical assays

Immediately after the animals were euthanized, the mucosa of each stomach was scraped using two glass slides, homogenized in a phosphate buffer (0.1 M, pH 7.4) and frozen at −80°C until it could be assayed biochemically. The protein concentration of the samples was determined by the method described by Bradford [5].

### 2.7. Effect of menthol on the activity of myeloperoxidase (MPO)

The MPO activity in the gastric mucosa was measured to evaluate the accumulation of neutrophils. The samples were centrifuged at 3,000×*g* for 15 min at 4°C. Aliquots of the supernatant were then mixed with a reaction buffer (pH 6.8) of 50 mM PBS in 0.005% H_2_O_2_ and 1.25 mg/mL O-dianisidine dihydrochloride (Sigma-Aldrich, St. Louis, MO, USA) and measured at 460 nm [6].

### Assays of antioxidant activity

### 2.8. Determination of total glutathione levels (GSH)

The GSH levels in the gastric tissue were determined by Ellman's reaction using 5′5′-dithio-bis-2-nitrobenzoic acid (DTNB, Sigma-Aldrich, St. Louis, MO, USA) [7]. The intensity of the yellow color was read using spectrophotometry at 412 nm.

### 2.9. Glutathione peroxidase activity (GSH-Px)

The GSH-Px activity was measured by combining 10 mM of reduced glutathione, 4 mM of NADPH, and 1 U enzymatic activity of GR (Sigma-Aldrich, St. Louis, MO, USA); the decrease in absorbance was induced by 0.25 mM H_2_O_2_ and monitored every minute for 10 min at 365 nm [8].

### 2.10. Glutathione reductase activity (GR)

The GR activity was measured by monitoring the decrease in absorbance of NADPH in phosphate buffer, pH 7.8 at 340 nm induced by oxidized glutathione (Sigma-Aldrich, St. Louis, MO, USA). The absorbance was read every minute for 10 min [9].

### 2.11. Superoxide dismutase activity (SOD)

The SOD activity was analyzed by the reduction of nitroblue tetrazolium using a hypoxanthine-xanthine oxidase system (superoxide generation, Sigma-Aldrich, St. Louis, MO, USA). The absorbance was read every minute for 10 min at 560 nm [10].

**Mediators of inflammation**

### 2.12. Determination of the gastric mucosal levels of TNF-α, IL-6 and IL-10

The tissue homogenate was centrifuged and the cytokines were detected in the supernatant using commercial enzyme-linked immunosorbent assay (ELISA) kits for TNF-α, IL-6 and IL-10 (catalog numbers 438204, 431304 and 431404, respectively, BioLegend, San Diego, CA, USA).

### 2.6. Statistical analysis

The results were analyzed using a one-way analysis of variance (ANOVA) followed by Dunnett's test and compared to the vehicle group. The results are presented as the mean ± the standard error of the mean (SEM). All analyses were performed using GraphPad InStat or Prism software. A value of p<0.05 was considered significant.

## Results

### 3.1. Ethanol induced gastric ulcers

**Figure 1:**
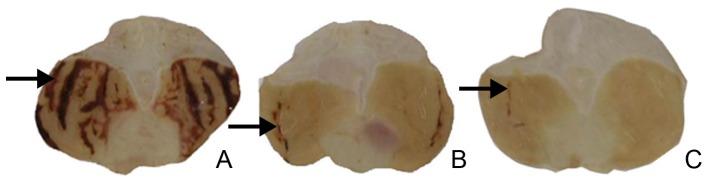
Effects of menthol on the gastric mucosa after ethanol induction of gastric ulcers in rats. Arrows indicate the characteristic necrotic bands forming the gastric ulcer. (A) Vehicle, (B) Carbenoxolone 100 mg/kg, (C) Menthol 50 mg/kg.

**Figure 2:**
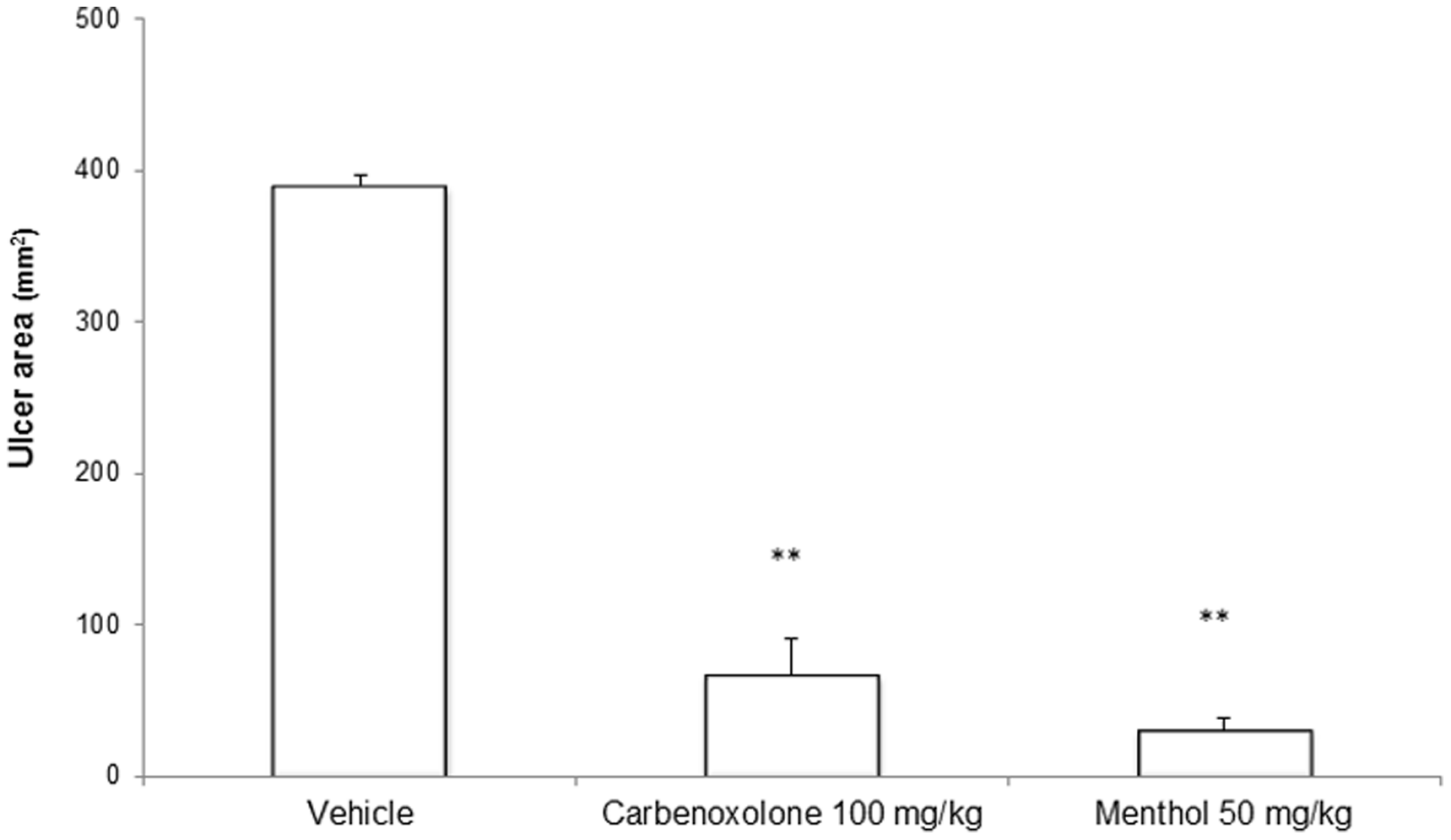
Gastric ulcer area (mm^2^) of rat stomachs with ethanol-induced gastric ulcers after treatment with vehicle, carbenoxolone (100 mg/kg) or menthol (50 mg/kg). The results are reported as the mean ± SEM. ANOVA followed by Dunnett's test, p<0.01.

The vehicle-treated group presented the characteristic necrotic bands in the gastric mucosa, with an average ulcer area of 389.79±6.46 mm^2^. The carbenoxolone-treated group presented an average ulcer area of 66.82±23.98 mm^2^, representing 83% gastroprotection, as defined by the average ulcer area. The menthol treated group showed a significant decrease (p<0.01) in the average ulcer area (30.34±8.04 mm^2^), corresponding to 92% gastroprotection. A representative stomach of each group is shown in Figure 1, and the ulcer areas are listed in Figure 2.

### 3.2. Histological analysis

The infiltration of neutrophils into the gastric submucosa was significantly decreased in the carbenoxolone- and menthol- treated groups (p<0.01) compared to the vehicle-treated group (Table 1).

**Table 1:** Neutrophils count and immunolabeled area (µm2) for HSP-70 and Bax in rats stomachs submitted to ethanol-induced gastric ulcers after treatment with vehicle, carbenoxolone (100 mg/kg) or menthol (50 mg/kg).

	Vehicle	Carbenoxolone	Menthol
Neutrophils count	23.40±0.62	13.60±1.65**	9.60±1.68**
Immunolabeled area for HSP-70	659.32±88.47	6547.01±984.22***	9620.86±544.65***
Immunolabeled area for Bax	11443.28±486.13	821.36±95.80**	199.36±26.84***

The results are reported as the mean ± SEM. ANOVA followed by Dunnett's test.

** p<0.01 or.

*** p<0.001.

### 3.3. Immunohistochemical analysis

**Figure 3:**
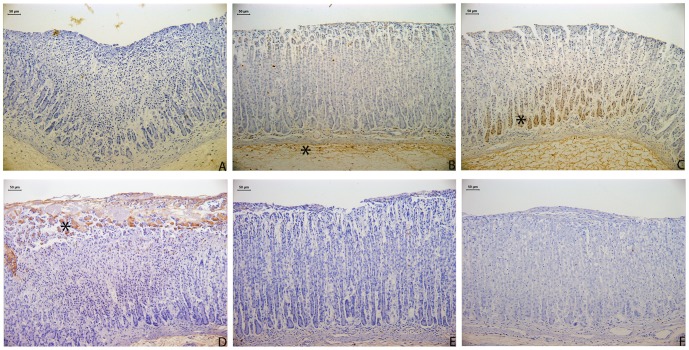
Photomicrography of the immunohistochemical analysis of HSP-70 expression (A, B, C) and Bax protein expression (D, E, F) in the stomachs of rats subjected to ethanol-induced gastric ulcers after treatment with vehicle (A, D), carbenoxolone 100 mg/kg (B, E) or menthol 50 mg/kg (C, F). The * in the brown stains indicates the in situ expression of the proteins. Note the upregulation of HSP-70 expression and the downregulation of Bax expression in the menthol-treated group.

Treatment with menthol caused an increase in the expression of HSP-70 in the menthol treated group compared to the vehicle-treated group; this was confirmed by an analysis of the HSP-70 immunolabeled area. Treatment with menthol also reduced the expression of the apoptotic protein Bax and decreased the immunolabeled area. These results are presented in Table 1 and Figure 3.

### 3.4. Effect of menthol on MPO activity

**Figure 4:**
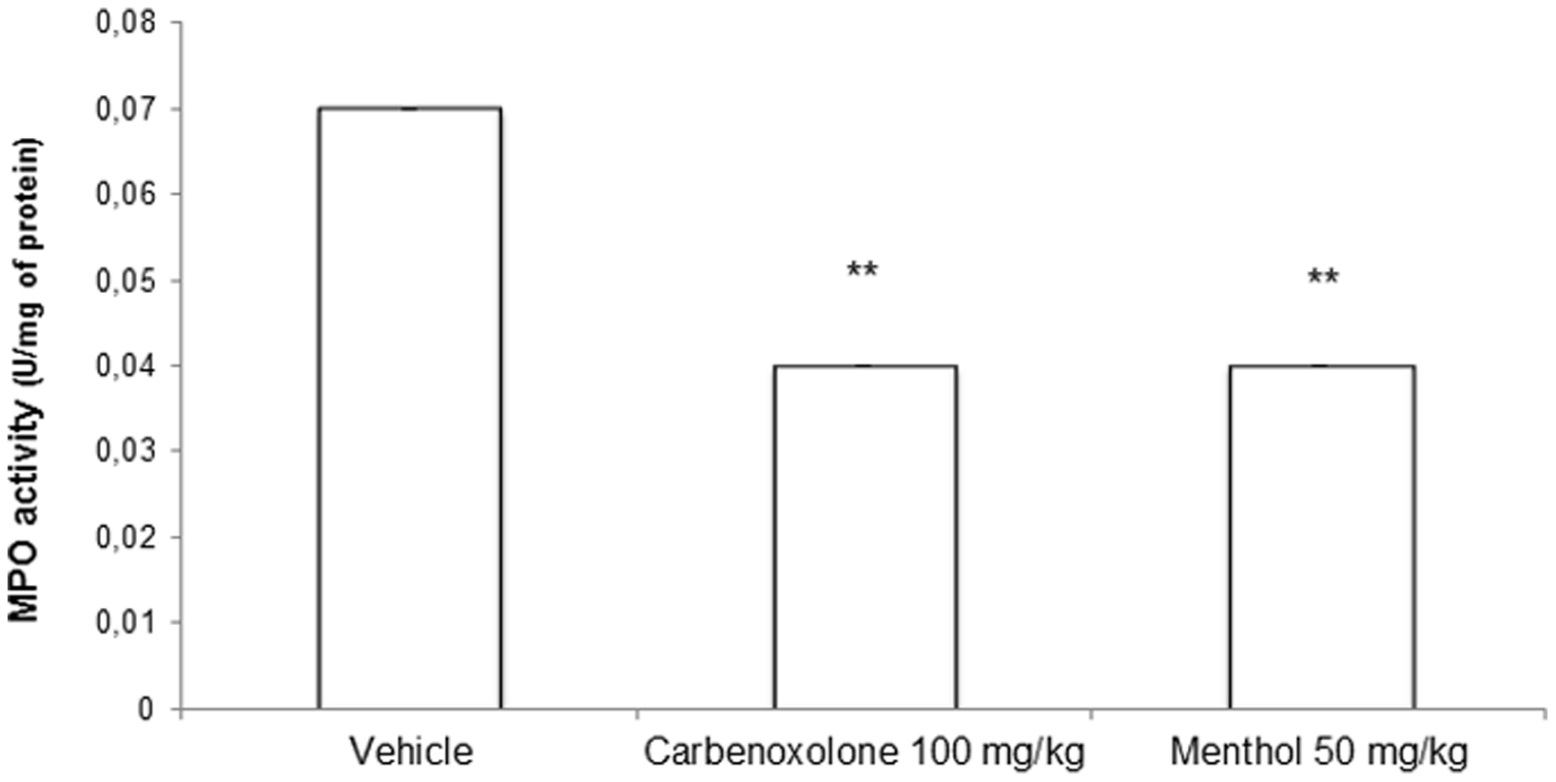
Myeloperoxidase (MPO) activity (U/mg of protein) in rat stomachs with ethanol-induced gastric ulcers after treatment with vehicle, carbenoxolone (100 mg/kg) or menthol (50 mg/kg). The results are reported as the mean ± SEM. ANOVA followed by Dunnett's test, p<0.01.

Treatment with menthol induced a significant decrease in the activity of the MPO compared to the vehicle-treated group (Figure 4); this result confirmed that ethanol administration induced the infiltration and activation of neutrophils in the gastric mucosa.

### 3.5. Antioxidant assays

Menthol showed an antioxidant activity, demonstrated by increases in the activities of the antioxidant compound GSH and the enzymes GSH-Px and GR in the menthol-treated group compared to the vehicle-treated group. The level of the SOD enzyme in the gastric tissue was decreased in the menthol treated group (Table 2).

**Table 2:** Effect of menthol (50 mg/kg) on the levels of antioxidant enzymes and compound in rat stomachs with ethanol-induced gastric ulcers after treatment with vehicle, carbenoxolone (100 mg/kg) or menthol (50 mg/kg).

	Vehicle	Carbenoxolone	Menthol
GSH	27.62±2.85	50.78±6.82**	60.87±6.00***
GSH-Px	96.07±7.21	108.30±14.67	171.30±13.15***
GR	32.64±1.99	40.95±3.12	48.80±3.34**
SOD	3.33±0.50	1.65±0.38*	1.97±0.26*

GSH level is expressed in nmol/mg of protein; GPx and GR are expressed in nmol/min/mg of protein; SOD is expressed in U/mg of protein. The results are reported as the mean ± SEM. ANOVA followed by Dunnett's test.

* p<0.05.

** p<0.01.

*** p<0.001.

### 3.6. Anti-inflammatory activity

Menthol demonstrated an immunomodulatory effect; menthol reduced the levels of cytokines in the gastric tissue, diminished the levels of the pro-inflammatory cytokines TNF-α and IL-6 and increased the level of the anti-inflammatory factor IL-10 (Table 3).

**Table 3:** Effect of menthol (50 mg/kg) on the levels of TNF-α, IL-6 and IL-10 cytokines in rat stomachs with ethanol-induced gastric ulcers after treatment with vehicle, carbenoxolone (100 mg/kg) or menthol (50 mg/kg).

	Vehicle	Carbenoxolone	Menthol
TNF-α	2076.00±122.80	1307.00±283.30*	144.70±59.04***
IL-6	1239.00±83.47	871.30±173.10	143.50±50.95***
IL-10	1718.00±344.10	4772.00±581.50***	3776.00±122.40***

The results are expressed as pg/mg of protein and reported as the mean ± SEM. ANOVA followed by Dunnett's test.

* p<0.05.

*** p<0.001.

## Discussion

Ethanol is commonly used to induce ulcers in experimental rats; ethanol leads to intense gastric mucosal damage, directly and indirectly through such mediators as reactive oxygen species (ROS) and cytokines [11]. In this study, the HSP-70-inducing, anti-apoptotic, antioxidant and anti-inflammatory activities of menthol in an ethanol-induced gastric ulcer model were investigated in rats. The dose of menthol used in this study (50 mg/kg) was selected based on previous results reported by our group showing the gastroprotective effect of menthol [3]. It was observed that treatment with menthol provided 92% gastroprotection compared to treatment with a vehicle control; this level of gastroprotection that is similar to the effect of the standard drug carbenoxolone (83%). The mechanisms responsible for the gastroprotective effect were also investigated.

HSPs are a type of protective protein involved in diverse biological activities, including apoptosis. HSP-70 is a molecular chaperone that is rapidly induced by stresses such as heat, oxidative stress, and drug exposure [12]. Therefore, drugs or treatments that induce HSP-70 expression may positively contribute to gastric mucosal defense and cytoprotection [13], [14]. In addition to its cytoprotective effect, HSP-70 has anti-apoptotic activity [15], [16] and can decrease oxidative stress and cell injury [14]. The induction of HSP-70 is part of the gastroprotective mechanism of menthol. Furthermore, the inhibition of apoptosis (observed as the inhibition of Bax expression) in the gastric mucosa of rats treated with menthol can be partially explained by menthol's HSP-70-inducing effect. It has been suggested that an interaction between the expression of the HSP-70 and the proapoptotic Bax genes may occur under stress conditions, with suppression of Bax activation in cells with high HSP-70 levels [17].

There is growing evidence that ethanol administration promotes oxidative stress by increasing the formation of ROS and depleting cellular oxidative defenses in a process triggered by neutrophil activation, causing a sequential ROS-mediated induction of lipid peroxidation and protein oxidation. Recent studies have linked the genesis of ethanol-induced gastric ulcers to the number of infiltrated neutrophils [18].

The MPO is the main marker of neutrophil infiltration in ulcerogenic lesions. This enzyme is found within the neutrophils and catalyzes the oxidation of the chloride ion (Cl^−^) by hydrogen peroxide (H_2_O_2_) to form hypochlorous acid (HClO), which is toxic to pathogenic microorganisms but is also harmful to host tissues [19]. This process is responsible for the generation of free radicals, resulting in an acute inflammation in the gastric tissue [20]. The gastroprotection induced by menthol can be partially explained by the inhibition of neutrophil infiltration and subsequent MPO generation.

Neutrophils also produce the superoxide radical anion (O_2_^−^), the product of the reaction of oxygen molecules and electrons from the transport chain in mitochondria. The cells of the gastrointestinal tract have an antioxidant defense system that is capable of preventing the cytotoxicity of ROS through mechanisms that involve the action of enzymes and compounds with the potential to scavenge free radicals and prevent their destructive action. The major antioxidative enzyme is SOD, which catalyzes the dismutation of O_2_^−^ into less noxious H_2_O_2_, which is further degraded by catalase or GSH-Px [21].

The activity of SOD was decreased in the stomachs of menthol- or carbenoxolone-treated rats. We propose that this is an interesting finding because the relatively higher SOD activity in vehicle-treated rats indicates a greater production of O_2_^−^ [22]. The increases in the amounts of the GSH-Px and GR enzymes and the antioxidant compound glutathione (GSH), associated with the inhibition of MPO production, confirm the antioxidant activity of the menthol in gastric ulcers and support an important role for oxidative stress in the pathogenesis of ethanol-induced gastric ulcers. There are several reports in the literature indicating that the administration of potential antioxidant natural products can prevent the gastric damage caused by the action of ethanol [23], [24], [25].

In generating ROS, the administration of absolute ethanol provokes an inflammatory response that is the result of a complex chain of events involving the immune response, which releases a great number of inflammatory cytokines such as tumor necrosis factor-α (TNF-α) and interleukin-6 (IL-6) [26].

Inflammation is a harmful process that should, in general, be minimized. However, in the gastrointestinal tract, an adequate inflammatory response is a key component of mucosal defense [27]. TNF-α is a pro-inflammatory cytokine that is increasingly secreted by macrophages during gastric ulcer induction [28]. It is a potent stimulator of neutrophil infiltration into the gastric mucosa [29]. The suppression of TNF-α production and neutrophil infiltration are closely associated [30]. IL-6 is a pleiotropic cytokine that plays a crucial role in acute inflammation and immune regulation [31]. An elevated level of IL-6 activates neutrophils, lymphocytes and monocytes/macrophages at the inflammatory site, triggering the oxidative pathways responsible for local tissue damage in gastric ulcer disease [32]. It was suggested that the pro-inflammatory cytokines TNF-α and IL-6 are important in regulating the severity of gastric ulcers [33]. The secretion of both cytokines enhances the effects of oxidative stress by inducing mitochondrial ROS generation and cytotoxicity [34].

IL-10 is one of the most important anti-inflammatory and immunosuppressive cytokines [35]. IL-10 suppresses the inflammatory response and inhibits the production of TNF-α [36], [37]. The results reported here demonstrate that the treatment with menthol induced a decrease in the levels of the pro-inflammatory mediators TNF-α and IL-6 and increased the level of the anti-inflammatory cytokine IL-10, demonstrating an anti-inflammatory activity of menthol.

## Conclusion

The treatment of ethanol-induced gastric ulcers in rats with menthol demonstrated that menthol exerts gastroprotection via an HSP-70-inducing effect, which leads to an anti-apoptotic effect through the inhibition of Bax production. Furthermore, menthol induces a decrease in the migration and activation of neutrophils as demonstrated by a decrease in MPO activity, which results in an antioxidant activity, an increase in the activities of GSH-Px and GR, an increase in the levels of GSH and a decrease in SOD activity. Menthol also induced an immunomodulatory and anti-inflammatory activity; menthol treatment decreased the levels of the pro-inflammatory cytokines TNF-α and IL-6 and augmented the levels of the anti-inflammatory cytokine IL-10.
